# Trends in TAVI

**DOI:** 10.1007/s12471-018-1141-1

**Published:** 2018-08-01

**Authors:** F. van Kesteren, J. J. Piek

**Affiliations:** 1 Heart Centre, Amsterdam University Medical Centre, AMC, Amsterdam, The Netherlands; 2 Department of Radiology and Nuclear Medicine, Amsterdam University Medical Centre, AMC, Amsterdam, The Netherlands

In the past decade transcatheter aortic valve implantation (TAVI) has become a life-saving, minimally invasive therapy for many patients with severe aortic valve stenosis. Initially TAVI was conceived as a last resort for inoperable patients or as an alternative for individuals at high surgical risk, but soon patients with an intermediate surgical risk followed. After the success of large randomised PARTNER trials, the number of procedures increased rapidly [1–3].

In this issue of the Netherlands Heart Journal, van Kesteren et al. describe the evolution of TAVI care in their hospital, where they performed more than 1,000 procedures over an 8‑year period [4]. From 2009 onwards, they found a shift towards a lower-risk population with less comorbidity. Along with the shift in the eligible population, they illustrate the evolution in the procedure itself, including the use of computed tomography angiography imaging for optimal sizing of the prosthesis, a reduction in delivery sheath size and the move to a minimalist approach under local anaesthesia. Operators and the institution gained more experience and, although only briefly mentioned in this article, TAVI devices were refined extensively over this 8‑year period. All this led to the most important results for clinicians and their patients: the improved clinical outcome over the years. The authors show that mortality after TAVI declined impressively, mainly as a consequence of improved 30-day clinical outcome including lower complication rates.

How do the described trends over the last 8 years of TAVI help us? And what can we tell about the future? Analysing such trends over time enables us to review the remaining limitations and important complications of TAVI. To further improve clinical outcome, it is important to study these complications carefully, for example by resolving the pathophysiology of stroke, which remains an invalidating major complication. Regarding this complication, the future will hopefully bring optimised pharmacotherapeutic and mechanical preventive strategies to reduce its incidence. Moreover, although the focus of the article in this issue is mostly on survival, for patients it is important that improved survival also yields in a good quality of life. Ideally, a risk score for potential TAVI candidates will provide a more accurate prediction of expected morbidity and mortality, which could help in patient selection. Perhaps computed tomography angiography can also play a larger role in this selection by including the evaluation of the cardiac function and coronaries in pre-TAVI scanning. Finally, although there is increasing evidence for the short-term safety of TAVI, and 5‑year outcomes look promising, more information on long-term outcomes and valve durability is required [5, 6]. To reach long-term valve durability, valve sizing and positioning must be optimal. Against this background, there are an increasing number of TAVI devices coming on the market besides the standard self-expandable and balloon-expandable prostheses. Nevertheless, there are only small trials evaluating prostheses quality [7]. Large randomised trials should provide an answer to the question of which prosthesis for which patient, and evaluate which patients are likely to benefit from the procedure and which patients would not benefit at all. Presumably, future improvements will result in further expansion of the clinical indications for TAVI and eventually it may even replace surgical valve replacement for most indications, but as the Nobel Prize winner Niels Bohr once said: “prediction is very difficult, especially if it is about the future”.
